# The Prognostic and Predictive Role of Xeroderma Pigmentosum Gene Expression in Melanoma

**DOI:** 10.3389/fonc.2022.810058

**Published:** 2022-01-31

**Authors:** Sarah Fischer, Mohamed Hamed, Steffen Emmert, Olaf Wolkenhauer, Georg Fuellen, Alexander Thiem

**Affiliations:** ^1^ Institute for Biostatistics and Informatics in Medicine and Ageing Research, Rostock University Medical Center, Rostock, Germany; ^2^ Department of Systems Biology and Bioinformatics, University of Rostock, Rostock, Germany; ^3^ Clinic and Policlinic for Dermatology and Venereology, Rostock University Medical Center, Rostock, Germany; ^4^ Leibniz-Institute for Food Systems Biology, Technical University of Munich, Freising, Germany

**Keywords:** melanoma, anti-PD-1, biomarker, DNA damage response, nucleotide excision repair, xeroderma pigmentosum, RNA-seq, gene expression

## Abstract

**Background:**

Assessment of immune-specific markers is a well-established approach for predicting the response to immune checkpoint inhibitors (ICIs). Promising candidates as ICI predictive biomarkers are the DNA damage response pathway genes. One of those pathways, which are mainly responsible for the repair of DNA damage caused by ultraviolet radiation, is the nucleotide excision repair (NER) pathway. Xeroderma pigmentosum (XP) is a hereditary disease caused by mutations of eight different genes of the NER pathway, or POLH, here together named the nine XP genes. Anecdotal evidence indicated that XP patients with melanoma or other skin tumors responded impressively well to anti-PD-1 ICIs. Hence, we analyzed the expression of the nine XP genes as prognostic and anti-PD-1 ICI predictive biomarkers in melanoma.

**Methods:**

We assessed mRNA gene expression in the TCGA-SKCM dataset (n = 445) and two pooled clinical melanoma cohorts of anti-PD-1 ICI (n = 75). In TCGA-SKCM, we applied hierarchical clustering on XP genes to reveal clusters, further utilized as XP cluster scores. In addition, out of 18 predefined genes representative of a T cell inflamed tumor microenvironment, the TIS score was calculated. Besides these scores, the XP genes, immune-specific single genes (CD8A, CXCL9, CD274, and CXCL13) and tumor mutational burden (TMB) were cross-correlated. Survival analysis in TCGA-SKCM was conducted for the selected parameters. Lastly, the XP response prediction value was calculated for the two pooled anti-PD-1 cohorts by classification models.

**Results:**

In TCGA-SKCM, expression of the XP genes was divided into two clusters, inversely correlated with immune-specific markers. A higher ERCC3 expression was associated with improved survival, particularly in younger patients. The constructed models utilizing XP genes, and the XP cluster scores outperformed the immune-specific gene-based models in predicting response to anti-PD-1 ICI in the pooled clinical cohorts. However, the best prediction was achieved by combining the immune-specific gene CD274 with three XP genes from both clusters.

**Conclusion:**

Our results suggest pre-therapeutic XP gene expression as a potential marker to improve the prediction of anti-PD-1 response in melanoma.

## Introduction

Immune checkpoint inhibitors (ICIs) are a standard treatment for advanced melanoma and other immunogenic tumors. For the therapy of melanoma, they include ipilimumab, a monoclonal antibody directed against the cytotoxic T-lymphocyte-associated antigen 4 (CTLA-4) receptor, and nivolumab or pembrolizumab, antibodies targeting programmed cell death-1 (PD-1) receptor (1–3). Despite the impressive and long-lasting clinical activity of ICIs in some patients, many do not respond. Furthermore, severe side effects are frequent, especially in the combined application of ipilimumab and nivolumab (4). These typically include immune-related adverse events of multiple organs and tissues, leading to inflammations such as thyreoiditis, pneumonitis, colitis or hypophysitis (1, 3, 4). Thus, predictive biomarkers of ICI response are urgently needed in order to identify those patients who achieve the greatest ICI benefit (1–3).

For the efficacy of anti-PD-1 ICIs, different predictive biomarkers have been proposed (5, 6). These can be classified as follows: *tumor-intrinsic* biomarkers (e.g., tumor mutational burden (TMB) or neoantigen load), which are indirect measures of tumor antigenicity generated by somatic tumor mutations, and *immune-specific* biomarkers (e.g., T cell-inflamed gene expression profiles (GEPs) or programmed-death-ligand 1 (PD-L1) expression), which are indicative of a T cell-inflamed tumor microenvironment (TME) (6, 7).

Particularly, many studies on immune-specific biomarkers have been conducted recently (8). For instance, Ayers et al. (9) analyzed GEPs using RNA from baseline tumor samples of patients treated with pembrolizumab and eventually defined an 18-gene GEP, hereafter referred to as the *Tumor Inflammation Signature* (TIS). This signature was predictive in 220 patients with nine different cancers and contained IFN-gamma–responsive genes related to antigen presentation, chemokine expression, cytotoxic activity, and adaptive immune resistance (9). In a current large-scale metanalysis of 1,008 ICI treated cases (n = 353 with melanoma), different predictive biomarkers of ICI response were compared with each other (10). In the markers of immune infiltration category, the TIS single genes *CXCL9*, *CD8A*, and TIS itself were the predictors with the strongest effect size. The gene *CXCL13* was also a highly predictive gene marker in the whole tumor cohort. Intriguingly, in the three melanoma anti-PD-1 cohorts (7, 11, 12) included in this meta-analysis, *CD274* (coding for PD-L1) was a further predictive marker. However, looking at each cohort individually, only in the cohort published by Cristescu et al. (7) TIS, *CXCL9*, and *CD274* were significantly positively associated with ICI response. Finally, the authors concluded that 34 predefined biomarkers (among them the markers of immune infiltration) could only explain about 60% of the total proportion of variance in ICI response, indicating that the remaining factors determining ICI response still need to be discovered (10).

Recent studies revealed that mutational processes directly altering the DNA damage response (DDR) could influence response to ICI (13–16). As one mechanism, DDR defects can lead to a higher TMB, which implicates a greater abundance of immunogenic neoantigens; this is impressively illustrated by the strong clinical activity of anti-PD-1 ICI in mismatch-mediated repair (MMR) deficient tumors (17–21). Notably, besides MMR, two other pathways are responsible for the repair of DNA single-strand breaks (SSB): base excision repair (BER) and nucleotide excision repair (NER). In contrast, DNA double-strand breaks (DSB) are repaired by homologous recombination and further by more error-prone nonhomologous end joining and microhomology-mediated end joining (22–25) Another DNA repair pathway is the Fanconi Anemia/BRCA pathway that restores DNA interstrand crosslinks.

In addition to an increased TMB, other more specific mechanisms leading to altered immunogenicity have been attributed to modified DDR pathways and signaling (6, 15, 25). These mechanisms include upregulation of PD-L1 expression by enhanced DDR signaling through SSB or DSB. Expression of PD-L1 is additionally increased by depletion of BRCA2, which is involved in homologous recombination, or by depletion of Ku70/80, a critical factor of nonhomologous end joining, and by BER reduction (26, 27). Importantly, increased PD-L1 expression after DSB and SSB was associated with the activation of STAT1, STAT3 and IRF-1, which are all part of the canonical interferon (IFN)-gamma-pathway (28). Additionally, for loss of interstrand crosslink repair function in breast cancer, an increased IFN-related gene expression, namely, the two critical mediators of CD4^+^ and CD8^+^ T-cell chemotaxis, *CXCL10* and *CCL5*, was discovered (29). Those and other cytokines are involved in T-cell inflammation, which is often a prerequisite for anti-PD-1 ICI response (30). Mechanistically, the crosstalk between immune and cancer cells within the TME, leading to PD-L1 upregulation on cancer cells, is the basis for the mode of action of anti-PD-1 ICI (31). These observations support the joint analysis of DDR pathway and immune-specific gene expression in the TME (32).

Although several case reports stated impressive anti-PD-1 ICI responses of patients with NER germline defects, and while some of them have identified a higher TMB, the further immunogenic impact by alterations of this DDR pathway is far less explored (13, 25, 33–37). Biallelic pathogenic variants in one of the seven NER genes coding for the so-called complementation groups, *XPA*, *ERCC3*, *XPC*, *ERCC2*, *DDB2*, *ERCC4*, *ERCC5*, the NER gene *ERCC1*, and the gene coding for XP variant, *POLH*, are the causes of the rare hereditary disease Xeroderma pigmentosum (XP) (38). They lead to an absent or inactivated protein and are hereafter referred to as the nine *XP genes*. The NER is mainly responsible for the repair of UV-induced DNA lesions and is divided into a global genome (GG) and transcription-coupled (TC) repair subpathway, which shares a common end section (39). XP patients under age 20 years have a 10,000-fold increased risk for non-melanoma skin cancer and a 2,000-fold increased risk for melanoma, making skin cancer the most common cause of death in this population (40). Hence, XP patients with skin tumors could benefit greatly from successful ICI treatment, requesting investigation of the role of these nine XP genes for ICI response.

A recent study correlated DDR pathway mutations irrespective of XP disease with overall survival of 1,661 ICI-treated patients and revealed that the NER pathway was predictive of ICI benefit—independent of TMB and tumor type. However, in 40,181 unique cancers, only 3.4% of melanomas possessed NER gene mutations (41). Moreover, Litchfield et al. found no predictive role of DDR pathway mutations for ICI response in seven different tumor types (10). An aspect, presumably limiting further the predictive role of DDR mutations, is that different genes in the same DDR pathway can unevenly affect the TME and the ICI response, as shown for BRCA1 and BRCA2 mutations (11, 42).

Based on the discussion above, we focused on analyzing the nine XP genes away from mutation data to gene expression data to investigate the predictive role of XP gene expression as an anti-PD-1 response marker in melanoma. Accordingly, *The Cancer Genome Atlas Skin Cutaneous Melanoma project* (TCGA-SKCM) (43) dataset, consisting of systemic treatment-naïve primary and metastatic melanoma samples, was used to identify two primary clusters of XP gene expression. We discovered that these were inversely correlated with the expression of TIS and single immune infiltration genes. In TCGA-SKCM, no significant negative correlations between XP genes and TMB were observed. Importantly, besides being predictive for the response to a specific treatment, biomarkers can also be prognostic by providing information about the patients overall cancer outcome, regardless of therapy (44). Because this can potentially interfere with their predictive value, we used TCGA-SKCM to analyze the prognostic role of different factors, and from the XP genes found only the expression of *ERCC3* to be prognostic. In contrast, expression of XP genes and clusters thereof could better predict response to anti-PD-1 ICs than well-established *immune-specific* biomarkers in two pooled clinical cohorts.

## Material and Methods

### Study Design

**Figure 1:**
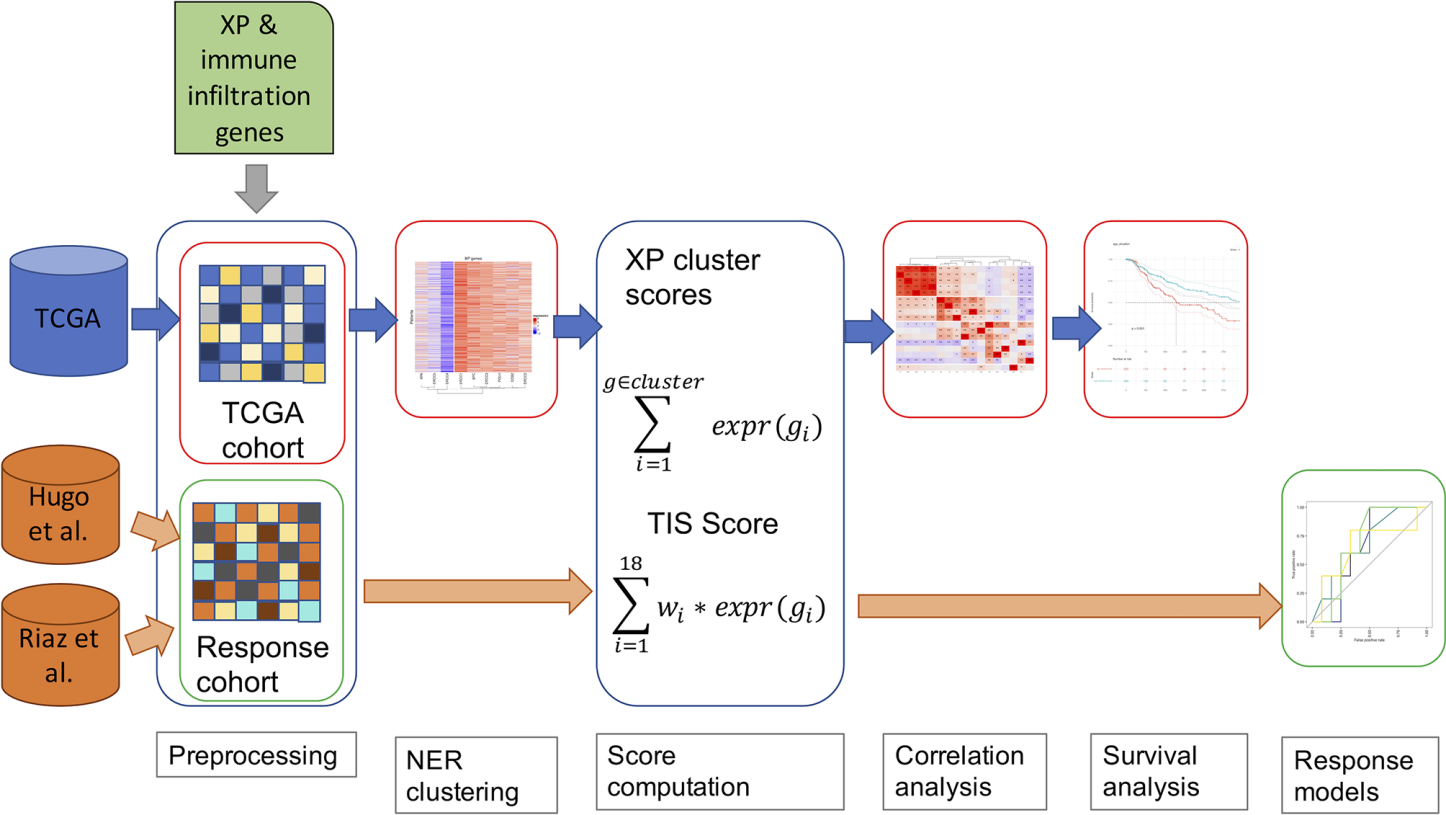
A schematic diagram of our workflow, including TCGA-SKCM and two anti-PD-1 cohorts of melanoma patients.

In **Figure 1**, we outline our analysis workflow beginning with the pre-processing of our three input datasets from TCGA-SKCM (43), Hugo et al. (11), and Riaz et al. (12). In parallel, we parsed the genes to be analyzed, the nine XP genes (*XPA*, *ERCC3*, *XPC*, *ERCC2*, *ERCC4*, *ERCC5*, *DDB2*, *POLH*, *ERCC1*), the 18 genes of the T- cell inflamed signature (9) (here named *Tumor Inflammation signature*, TIS) and the predictive biomarker, *CXCL13*, were retrieved through literature research (6, 10). The further utilized TIS score was calculated as the weighted sum of the 18 gene expression values according to Ayers et al. and Cristescu et al. (7, 45).

Accordingly, the XP gene expression in TCGA-SKCM was clustered hierarchically to define two XP clusters consisting of the mean expression of the corresponding genes. As an additional parameter, we included the TMB for the TCGA-SKCM data in our workflow. An underlying association was investigated *via* Spearman correlation between the computed scores, the particular gene expressions of XP genes, and predictive biomarkers and TMB. Afterwards, we assessed in TCGA-SCKM whether XP or TIS score, single XP or immune infiltration gene expression, or TMB could be prognostic for survival. Except for the pre-processing, the specified workflow was repeated for multiple sample subgroups split by clinical parameters such as age, sample type or gender.

To evaluate the potential of the XP genes as a predictive biomarker for the ICI response, we utilized the two anti-PD-1 datasets and developed simple prediction models using Youden’s index and Xtreme gradient boosting.

All data analyses were performed using R version 3.6.3 (46). A *p*-value <0.05 was considered statistically significant in all analyses, and a *p*-value <0.005 was highly statistically significant.

### Data Collection and Preprocessing

>Gene expression data used in this manuscript were obtained from TCGA-SKCM (http://cancergenome.nih.gov/, n = 464) (43) and two datasets of anti-PD-1 ICI cohort studies in melanoma patients, Hugo et al. (n = 28, GEO: GSE78220) (11) and Riaz et al. (n = 110, GEO: GSE91061) (12). The TCGA-SKCM dataset was reduced to n = 445 samples, which are fully annotated with clinical information, such as age, gender, and survival time. Likewise, we included samples of the other datasets after filtering for the mentioned clinical data availability and exclusion of on-treatment samples from the ICI cohorts, resulting in n = 26 (11) and n = 49 (12). All analyzed RNA-seq data were formatted as FPKM and log_2_ transformed. For TCGA-SKCM, somatic mutations were obtained from the TCGA data portal, and the TMB was calculated as log_10_ of the number of non-synonymous mutations per 50 Mb (package “*maftools*” v.2.2.10) (47). Responder (complete response [CR] or partial response [PR]) and non-responder (stable disease [SD] or progressive disease [PD]) were defined by RECIST criteria-based radiological response (7, 10). The clinical characteristics plus the scope of the computed scores of the utilized cohorts are listed in **Table S1**.

### Clustering

In the process of clustering method selection, multiple clustering methods and distance metrics of hierarchical clustering were tested (**Table S2**). Clusters containing only one single gene were excluded because singe genes analysis of XP genes was performed apart. Hence, as the final XP clusters we selected the best performing partition with at least two genes per cluster, which was supported by the majority of all tested clustering methods and distance metrics.

### Calculation of Scores

To identify a T cell-inflamed TME, we followed Ayers et al., based on the log_2_ transformed FPKM values; the TIS score was calculated as the weighted sum of the expression values of the 18 genes, enumerated in **Table S3**, applying the predefined weights derived by Ayers et al. (7, 9, 45). Considering the generation of scores based on the sum of signature related genes, we accordingly defined two XP gene cluster scores by summing up the expression values of genes in the same cluster.

### Correlation Analysis

To assess the co-expression relationship between the considered genes, we cross-correlated the specified parameters. The Spearman rank correlation with *p*-value adjustment using Benjamini–Hochberg was performed by R package “*psych*” (v.2.1.3) (48) and visualized with package “ComplexHeatmap” (v.2.2.0) (49) using complete clustering with Euclidean distance for the dendrogram displayed at the columns.

### Survival Analysis

For the survival analysis of the TCGA-SKCM data, we defined the overall survival (OS) as the time between melanoma diagnosis and the death or the last follow-up of the patient. The median follow-up was 669.50 days, while the survival status was decoded by 0 (alive) and 1 (dead). The constructed univariant Cox regression model predicted the overall survival from the continuous scores and gene expression values obtained from R packages “*survival*” (v.3.2-11) (50, 51) and “*survminer*” (v.0.4.9) (52). Kaplan–Meier analysis was used to calculate the survival probability of stratified patients, and the log-rank *p*-values for each analysis were given.

### Response Prediction Model Construction

For anti-PD-1 response analysis, expression data of the two clinical cohorts (11, 12) were downloaded and reanalyzed using the Wilcoxon test and comparing expression levels of scores and single genes between responder and non-responder samples. The Youden index with associated ROC was determined for each parameter with R package “*cutpointr*” (v.1.1.0) (53, 54). The analysis was extended by multivariable predictive models for classification with the machine learning algorithm XGBoost (v.1.4.1.) (55) by partitioning the samples 75%/25% to training and testing data, respectively. The performed classification into responder and non-responder used “*caret*” (v. 6.0-86) (56) with the “xgbTree” method (55) and 10-fold cross-validation for combinations of multiple parameters.

## Results

### Heterogeneity of XP Gene Expression in TCGA-SKCM

**Figure 2:**
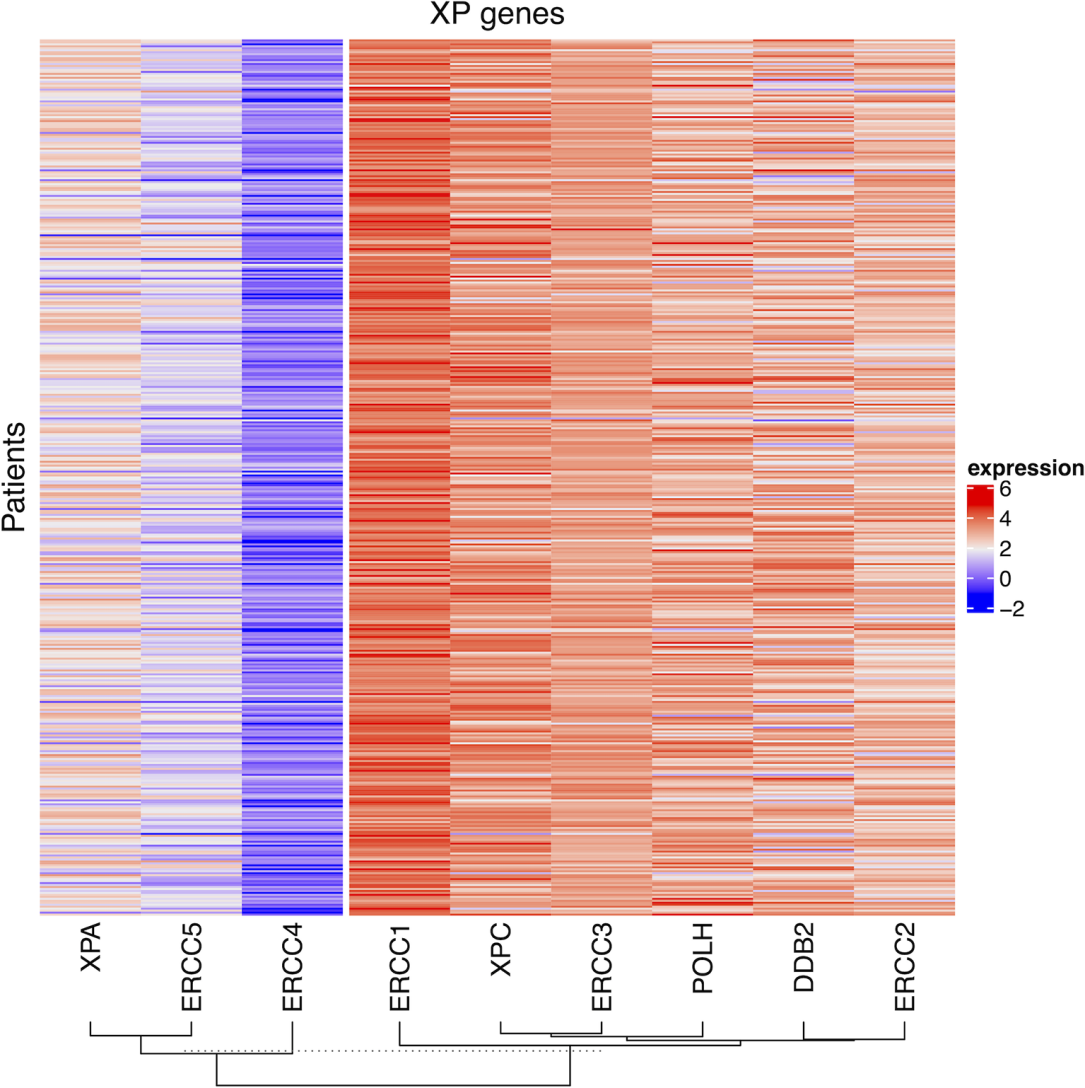
The heatmap of log_2_ transformed FPKM values of the nine XP genes for all patient samples in TCGA-SKCM. The columns are clustered by hierarchical clustering with Manhattan distance and complete linkage.

First, we explored the nine XP genes *XPA*, *ERCC3*, *XPC*, *ERCC2*, *ERCC4*, *ERCC5*, *DDB2*, *POLH*, and *ERCC1* in TCGA-SKCM and observed heterogeneous expression patterns. By unsupervised clustering, we could identify two XP gene expression clusters, referred to as XP gene clusters 1 and 2 (**Figure 2**). Cluster 1 comprised the genes *XPA*, *ERCC4*, and *ERCC5*, while cluster 2 included *ERCC3*, *XPC*, *ERCC2*, *DDB2*, *POLH*, and *ERCC1*. Remarkably, the same clustering appeared if the cohort had been priorly divided by sample type (primary or metastatic, **Figures S1A, B**), age (younger or older than median age of 58, **Figures S1C, D**), or gender (male or female, **Figures S1E, F**). Genes of both XP clusters and their function in the NER pathway and of *POLH* are summed up in **Table 1**.

**Table 1:** XP genes with corresponding clusters, their encoding proteins and their functionality in the NER pathway and translesion synthesis, respectively.

Genes	Cluster Membership	Corresponding Proteins	Main Function
*XPA*	1	XPA	Involved in multiple NER steps, e.g., DNA damage verification; interacts with almost all other NER proteins
*ERCC4*	1	XPF	DNA lesion excision in a complex with ERCC1 at 5ʹ end from the lesion
*ERCC5*	1	XPG	DNA lesion excision at 3ʹ end from the lesion
*ERCC3*	2	XPB	DNA damage verification as TFIIH basal transcription factor complex DNA helicase subunit
*XPC*	2	XPC	DNA-damage recognition in GG-NER
*ERCC2*	2	XPD	DNA damage verification as TFIIH basal transcription factor complex DNA helicase subunit
*DDB2*	2	XPE	Auxiliary DNA-damage-recognition factor in GG-NER
*POLH*	2	XPV	DNA polymerase η, which is an enzyme of translesion synthesis, that bypasses unrepaired DNA damage
*ERCC1*	2	ERCC1	DNA lesion excision in a complex with XPF at 5ʹ end from the lesion

GG, global genome; NER, nucleotide excision repair; TFIIH, transcription initiation factor IIH.

Altogether, median XP gene expression did not vary significantly in the analysis of subgroups. However, *ERCC4*, *XPC*, and *POLH* were expressed substantially greater in metastatic samples, whereas *DDB2* was expressed considerably higher in primary tumors. Subdividing the whole TCGA-SKCM cohort by median age, we found that in melanoma tissue from younger patients, XP cluster 1 genes and also *XPC* and *DDB2*, belonging to XP cluster 2, were expressed to a relatively higher extent (**Table S4**). In samples from female patients, all XP genes, except *ERCC1* and *ERCC2*, were expressed to a greater extent than in males.

### Correlation Analysis Between XP & Immune Infiltration Genes, TMB and Computed Scores

**Figure 3:**
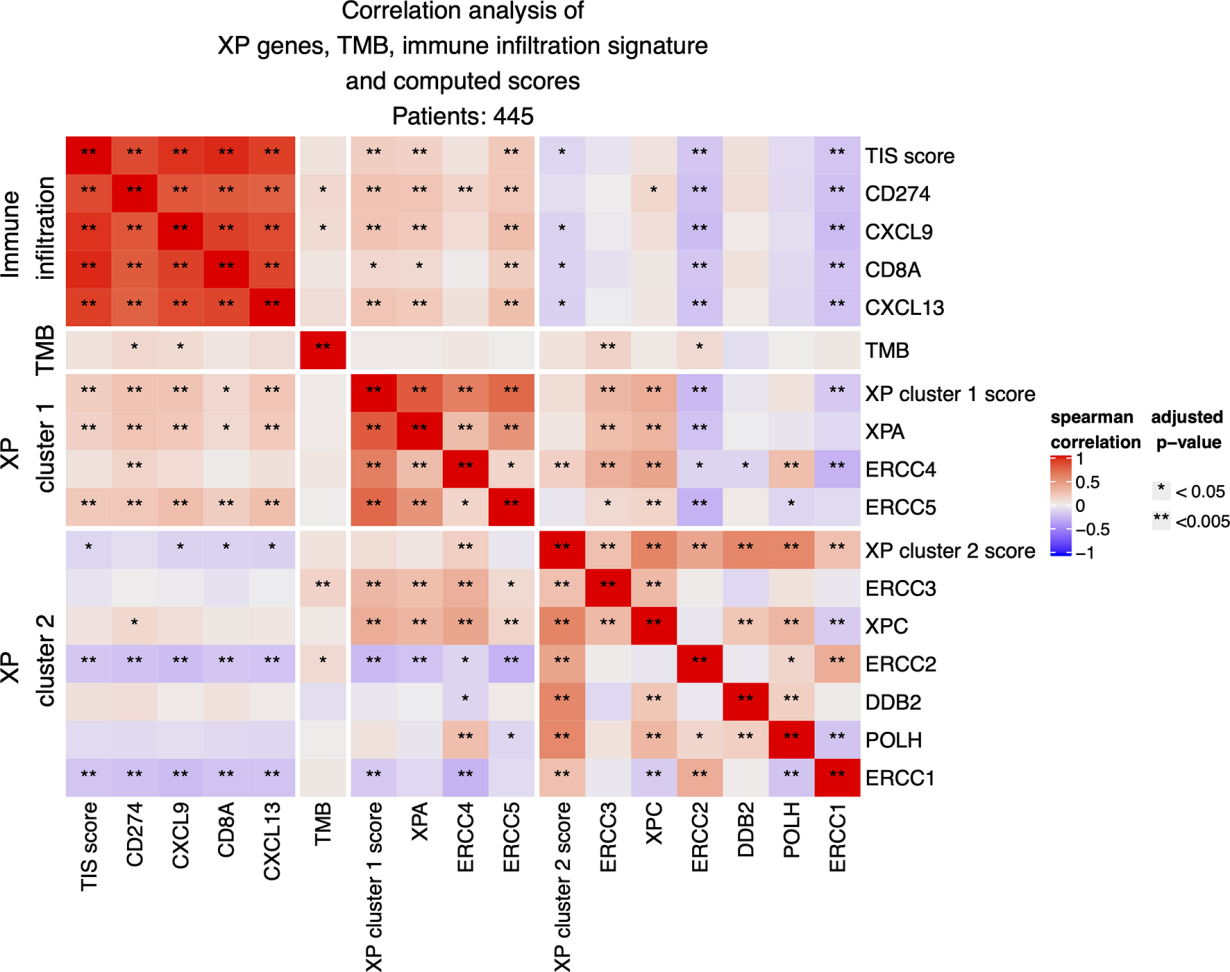
Correlation analysis between expression of the nine XP genes, of computed XP and TIS scores and of single immune infiltration genes.

Next, we investigated the correlation of XP genes and associated XP expression clusters to well-established predictive biomarkers of anti-PD-1 ICI response (**Figure 3**).

The expression of XP cluster 1 score with the 18-gene immune infiltration TIS score (*p* = 0.00034; R = 0.1793) as well with its single genes *CD274* (*p* = 6.858 e−07; R = 0.244), *CXCL9* (*p* = 1.640 e−06; R = 0.237), *CXCL13* (*p* = 2.666 e−06; R = 0.232), and *CD8A* (*p* = 0.0107; *R* = 0.131) showed weak but significant positive correlations. Likewise, the XP cluster 1 genes *XPA* and *ERCC5* were significantly positively correlated with TIS score and the above-mentioned immune infiltration genes. However, expression of *ERCC4* was only significantly correlated with *CD274* (*p* = 0.004; R= 0,146). Expression of XP cluster 2 score, on the other hand, was negatively correlated with the expression of TIS score (*p* = 0.037; R = −0.108), *CXCL9* (*p* = 0.013; R = −0.127), *CD8A* (*p* = 0.012; R = −0.129), and *CXCL13* (*p* = 0.01; R = −0.133). Importantly, the XP cluster 2 score genes *ERCC1* and *ERCC2* were individually highly significantly negatively correlated with TIS score (*p* = 0.0002, R = −0.186 and *p* = 0.0004, R = −0.176), immune infiltration genes and the XP cluster 1 score (**Figure 3**).

Tumor mutation burden (TMB) had weak positive correlations only with *CD274* (*p* = 0.01342; R = 0.127), *CXCL9* (*p* = 0.038; R = 0.1078), and also with the XP cluster 2 genes *ERCC3* (*p* = 0.00196; R = 0.157) and *ERCC2* (*p* = 0.027; R = 0.114). Importantly, no significant negative correlations between XP genes and TMB were observed.

When considering primary and metastatic samples separately, some differences were evident (**Figures S2A, B**): in primary samples only (n = 96), XP cluster 1 and cluster 2 scores had a positive correlation (*p* = 0.017; R = 0.279). Furthermore, the expression of the genes *ERCC3* and *XPC* was closely correlated with the expression of the XP cluster 1 score and its genes *XPA*, *ERCC4*, and *ERCC5*. Now, with a few exceptions, there were no significant correlations between XP genes and TIS score or immune infiltration genes but a positive correlation of TMB with TIS score (*p* = 0.00993; R = 0.3), *CD274* (*p* = 0.0048; R = 0.323), *CXCL9* (*p* = 0.0046; R = 0.327), *CD8A* (*p* = 0.004; R = 0.325), and *CXCL13* (*p* = 8.56 e−05; R = 0.422). Correlation of the by far larger group of metastatic samples (n = 349) revealed almost the same picture as for the whole group.

Further splitting by age and gender led to identical correlation patterns of XP gene clusters 1 and 2 with TIS score and immune infiltration genes, as we had observed for the whole TCGA-SKCM cohort (**Figures S2C–F**). Of note, the significant positive correlation of TMB with *CD274* was only detected if considering just males or the younger subgroup of patients.

### XP & Immune Infiltration Genes, TMB, and Computed Scores as Prognostic Biomarkers for Survival

The great majority of TCGA-SKCM samples were obtained in the pre-ICI era. Only two patients received anti-PD-1 ICI after acquiring their tumor, but the removal of these two patients did not lead to significantly different results (**Table S5**) (43). Hence, we sought to analyze if there is a linear association between the expressions of the XP genes or cluster scores with survival of patients in TCGA-SKCM, and independent of ICI. Additionally, we analyzed the prognostic value of parameters predictive of anti-PD-1 ICI response: The TIS score, selected single score genes (*CD274*, *CXCL9*, *CD8A*), *CXCL13* TMB, and age.

**Figure 4:**
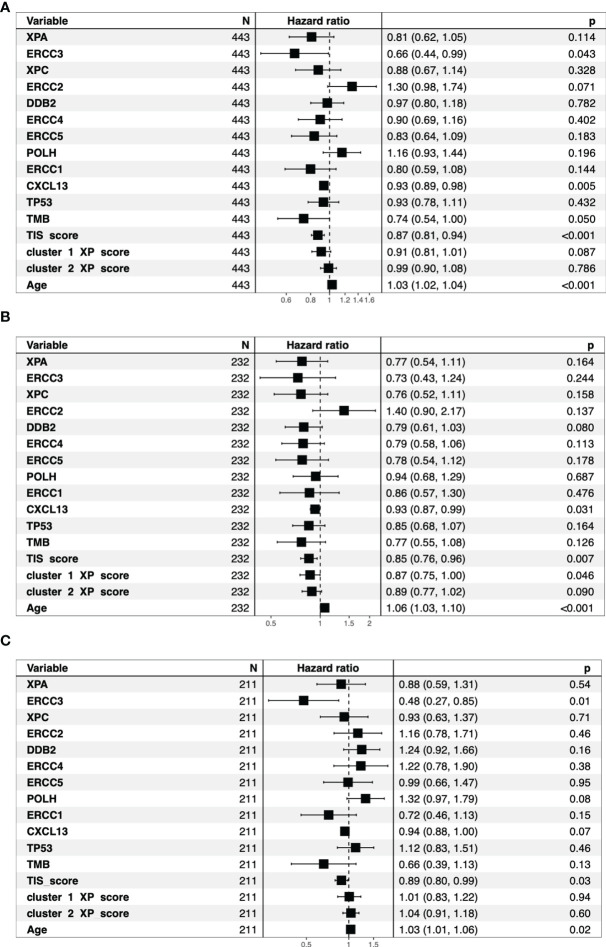
Overview of the univariate cox regression analysis for all TCGA-SKCM patients **(A)**. The bar indicates the reference Hazard ratio of 1. The patients split by median age into older patients **(B)** and younger patients **(C)** show different Hazard ratios for the same parameters.

**Figures S3C, D** demonstrated that neither single XP cluster 1 nor cluster 2 score were associated with survival in TCGA-SKCM. Considering the univariate Cox regression, out of the single XP genes, only *ERCC3* expression (hazard ratio, HR = 0.66, *p* = 0.043) revealed a significant association with survival (**Figure 4A**). In contrast, TIS score (HR: 0.87), *CXCL13* (HR = 0.93), TMB (HR = 0.74), and age (HR = 1.03) were all prognostic. TIS score and age also remained significant after segregation by the median. **Figures S3A, B** illustrate the corresponding Kaplan–Meier survival curves (log ranked *p*-values for age, *p* = 0.0014; TIS score, *p* = 0.0077).

The same analysis was repeated independently for age-divided sub-cohorts to decrease the influence of age as a dominant factor. Through this fractioning, the median overall survival dropped from 3,424 days to 1,927 days in the older patient group, while it increased to 4,634 days for the younger patients.

In those subgroups, we noted some differences (**Figures 4B, C** and **Figures S4**–**S7**). Although age and TIS score remained significant prognostic parameters, *ERCC3* (HR = 0.48, *p* = 0.0084) was the best predictor for survival and the only prognostic factor in the younger cohort. In the subgroup of older patients, *CXCL13* (HR = 0.93) and, newly, XP gene cluster 1 (HR = 0.87) were additional prognostic parameters.

### XP & Immune Infiltration Genes and Computed Scores as Predictive Biomarkers for Response to Anti-PD-1 ICI

Because of the remarkable anecdotal benefit of XP patients to anti-PD-1 ICI, we analyzed XP gene expression as a predictive biomarker for response to anti-PD-1 ICI in two pooled publicly available melanoma cohorts (n = 75) (11, 12).

**Figure 5:**
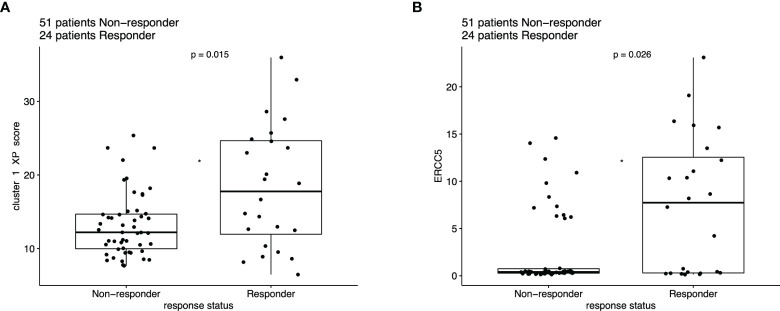
Boxplot of ICI response data (n = 75), compared with Wilcoxon test based on the expression of **(A)** XP cluster 1 score and **(B)** *ERCC5*.

The distribution of the responding (complete or partial response) patients differed significantly from the non-responders based on the XP cluster 1 score (*p* = 0.015), with a higher score indicating a greater response (**Figure 5A**). A similar significant difference between these two groups was also applied for the single XP cluster 1 gene *ERCC5* (*p* = 0.026) (**Figure 5B**). Importantly, XP cluster 2 score, TIS score, and single genes indicative of immune infiltration, except *CD27* and *PSMB10*, were not significantly associated with response in the pooled anti-PD-1 melanoma cohorts (**Figure S8**).

To assess the predictive performance of these single genes for ICI response, we computed the Youden index for each parameter (a XP gene or signature) and compared the areas under the receiver operating characteristic curves (AUC) (**Table S6**) (7). In this analysis, *ERCC5* (AUC = 0.660), XP cluster 1 score (AUC = 0.654), XP cluster 2 score (AUC = 0.632), and *CD8A* (AUC = 0.627) were the best performing variables. Except *XPA* (AUC = 0.532), *POLH* (AUC = 0.545), and *CD274* (AUC = 0.533), all other parameters outperformed TIS score (AUC = 0.586).

**Figure 6:**
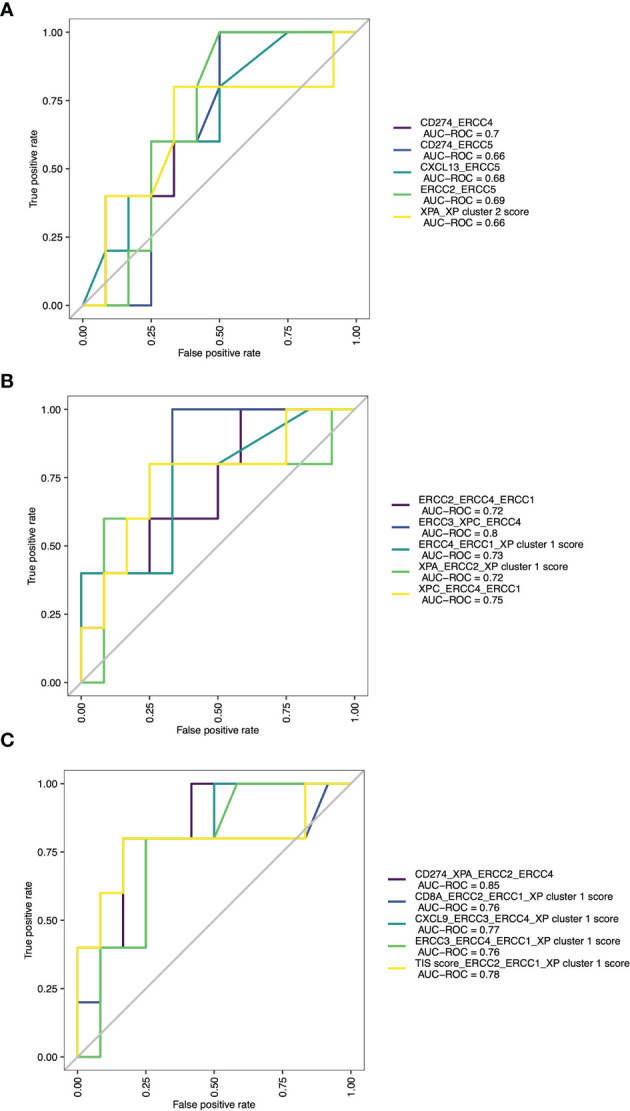
ROC curves with AUCs of top 5 combinations of **(A)** 2 parameters, **(B)** 3 parameters and **(C)** 4 parameters for prediction of ICI response.

Expansion of these restricted single parameter analyses by combining multiple variables from two to five possible parameters led to many response classification models. Due to infinitive values for two samples, this analysis was limited to 73 patients. The combination of two parameters had the best results if one immune infiltration gene (like *CD274* or *CXCL13*) was combined with one XP gene (CD274_*ERCC4*, AUC = 0.7; CXCL13_ERCC5, AUC = 0.68), or if XP gene cluster 2 gene *ERCC2* was combined with *ERCC5* (AUC 0.69). All these combinations outperformed any combination involving the TIS score (**Table S7** and **Figure 6A**). The three-parameter-based analysis performed better than the combination of two parameters and revealed that combining XP genes provided the best classification triplet for response (*ERCC3_XPC_ERCC4*, AUC = 0.8; *XPC*_*ERCC4*_*ERCC1*, 0.75). (**Table S8** and **Figure 6B**). The prediction of the combination of four variables had the best AUC value of 0.85, and even outperformed the combination of five parameters (**Tables S9**, **S10** and **Figure 6C**). Of note, it included the combination of one immune infiltration gene (*CD274*), two XP cluster 1 genes (*XPA*, *ERCC4*) and one XP cluster 2 gene (*ERCC2*).

## Discussion

Clustering by using mRNA gene expression levels can identify higher-level structures and relationships and establish a new molecular classification of tumors (57, 58). Furthermore, hierarchical clustering based on gene expression profiles (GEPs) can be used to, e.g., reveal immune competency or sensitivity to ICI treatment (9, 59).

Because melanoma has been the “model tumor” for the development of ICI and was also the first tumor entity in which ICI was approved, we focused our exploratory analysis on melanoma. Our study analyzed XP gene expression and deduced two different expression clusters (XP gene clusters 1 and 2, see **Figure 2** and **Table 1**) with heterogeneous functions in the NER pathway and translesion synthesis. The proteins encoded by the XP cluster 1 genes *ERCC4*, i.e., XPF, and *ERCC5*, i.e., XPG, are responsible for the dual incision of DNA damage. Cluster 1 further includes XPA, the central NER coordinator, because of its interaction with almost all other NER factors (39, 60). Accordingly, it also interacts with ERCC1, an endonuclease and fulfills its function as a heterodimer with XPF (61, 62). Surprisingly, *ERCC1* is part of XP cluster 2 instead of cluster 1, and there was a strong negative correlation between *ERCC1* and *ERCC4* expression in the TCGA-SKCM samples (**Figure 3**). Besides, in NER, the ERCC1–XPF complex is involved in interstrand crosslink and DSB repair, and mutations in one of the two genes result in a very complex constellation of clinical symptoms (39, 63). In epithelial ovarian cancer, ERCC1 and ERCC4 expression correlated on mRNA and protein level with one another, however, *ERCC1* mRNA was negatively correlated, and *ERCC4* mRNA was unrelated with its protein expression, suggesting a posttranscriptional mode of regulation (64). Because protein expression data was unavailable for most of our nine XP genes, we could not expand our analysis to protein correlations, which might have revealed substantially different clusters due to extensive posttranslational modifications in NER (39). In global genome-NER, damage recognition is performed by XPC and involves XPE (encoded by *DDB2*), which are both parts of XP cluster 2. Different genes of cluster 2 include *ERCC2* (codes for XPD) and *ERCC3* (codes for XPB*)*, which are DNA helicases and part of transcription initiation factor IIH complex verifying DNA damage lesions. *POLH*, whose defect leads to XP variant, codes for the DNA error-prone polymerase eta involved in translesion synthesis (39, 65).

Subsequently, we investigated the correlation of the XP genes and clusters with well-known, ICI predictive biomarkers of a T cell-inflamed TME: The TIS score, namely, its single genes *CD8A*, *CXCL9*, *CD274*, and the recently postulated biomarker *CXCL13*, that could be characteristic of clonal neoantigen-reactive CD8 T infiltrating lymphocytes (6, 7, 9, 10). Of note, we revealed that XP cluster 1 and its genes *XPA* and *ERCC5* had a highly significant positive correlation with the TIS score and all other immune infiltration associated genes (**Figure 3**). In line with this, Boonstra et al. (66) compared UVB suppression of ConA-induced IFN-γ production in XPA, XPC, and CSB deficient mice and demonstrated that only XPA mice showed a substantial reduction of IFN-γ production by UVB. Regarding gene correlations in TCGA-SKCM, our results are different from those of BER/SSB repair genes, which more homogeneously and almost exclusively present a negative correlation with *CD274* expression (26).

A negative correlation with *CD274*, TIS score, or the other genes representative of immune infiltration was identified for the XP cluster 2 genes *ERCC1* and *ERCC2*, which was true especially for samples of younger patients. However, neither the expression of *ERCC1*, *ERCC2*, nor of other XP genes was negatively correlated with TMB. Likewise, the frequency of XP gene mutations with median TMB values was not correlated in different cancers (41). Noteworthy, from the XP genes, *ERCC3* expression even had a highly significant positive correlation with TMB in samples of the whole cohort and the younger subcohort (**Figure 3** and **Figure S2C**). Taken together, the results from Hsiehchen et al. (41) and ourselves illustrate that the implications for tumor immunogenicity through XP gene mutations and diverse expression are presumably more complex than being based solely on the more abundant generation of neoantigens caused by somatic tumor mutations. Admittedly, we cannot precisely determine if the XP gene expression we assessed is preferentially constituted by tumor or other, e.g., immune cells in the TME (67). Circadian XP gene expression particularly affects *XPA* and could potentially impact the results of our analysis; however, it seems not to be relevant in actively proliferating tissues as tumors (68, 69).

Just recently, survival prognosis in melanoma was correlated with immune-related gene signatures (70–72). Of note, Danaher et al. (73) found that TIS was also highly statistically significantly prognostic in TCGA-SKCM, limiting its predictive value in melanoma patients. Accordingly, we analyzed the predictive role of all markers and found out that besides TIS, *CXCL13*, TMB, age, and *ERCC3* were prognostic. Age and TMB have been revealed as prognostic biomarkers in different studies and cancer entities before, though results for TMB are not homogeneous and depend on the used thresholds (74–77). Correspondingly, in our univariate analysis with segregated parameters for high and low values based on medians, we found no difference between TMB groups (**Table S11**). *CXCL13* was suggested as a prognostic biomarker in melanoma before, but the correlation of *ERCC3* with survival needs further validation (78, 79).

Notably, after dividing the cohort by age, in younger patients, *ERCC3* (HR 0.48) and TMB (0.66) revealed the lowest HR values; however, for TMB, it was not significant. This observation, together with the positive correlation between *ERCC3* and TMB in younger patients, suggests that *ERCC3* might have specific relevance for disease progression, especially in the young. In contrast, earlier reports have suggested that the presence of an intense immune infiltrate in older persons could have more prognostic weight (75). This assumption might explain that in our analysis, *CXCL13* expression, as a specific marker of exhausted T cells, was only prognostic in melanoma tissue of older when subdividing by patient age. The same accounted for XP cluster 1, which in samples of older was highly correlated with TIS score and other immune infiltration genes (**Figure 4** and **Figure S2D**).

An ongoing challenge is the identification of reliable biomarkers predictive of ICI response. Recent efforts leave single parameters and evolve into combinatorial biomarkers (6, 7, 10). Especially, the combination of tumor intrinsic factors, like TMB, and genes representative of immune infiltration in the TME, like TIS or *CXCL13*, show promise in exploratory studies (7, 9, 10, 80). Hence, we tested the single and combinational predictive performance of our parameters in two pooled anti-PD-1 cohorts. The existing *immune-specific* biomarkers were only of limited value and constructed prediction models (10). Importantly, we observed that the combination of either an immune-infiltrating gene (*CD274*) with three XP genes, or the combination of only three XP genes from both clusters provided the best ROC and AUC values (**Table 2**). Of note, the prediction was not improved by extension to five parameters; hence, we did not test further combinations of even more variables. Because of our small, pooled cohort and to avoid overfitting, we did conduct only a split of our data in training and testing set for our prediction models. We, therefore, admit that our constructed models lacked robustness to establish new ICI predictive biomarkers.

**Table 2:** Combination of the best 25 performing models, based on the AUC values, across the different number of used parameters.

Parameter for predictive model	AUC	# parameters
*CD274*_*XPA*_*ERCC2*_*ERCC4*	0.85	4
*ERCC3*_*XPC*_*ERCC4*	0.80	3
TIS score_*ERCC2*_*ERCC1*_cluster 1 XP score	0.78	4
TIS score_*CXCL13*_*CXCL9*_*CD274*_cluster 1 XP score	0.78	5
*CXCL9*_*CD8A*_*CD274*_*ERCC2*_cluster 1 XP score	0.78	5
*CXCL9*_*CD8A*_*XPC*_*ERCC1*_cluster 1 XP score	0.78	5
*CD8A*_*CD274*_*ERCC3*_*ERCC4*_cluster 1 XP score	0.78	5
*XPA*_*ERCC2*_*ERCC1*_cluster 1 XP score_cluster 2 XP score	0.78	5
*CXCL9*_*ERCC3*_*ERCC4*_cluster 1 XP score	0.77	4
*CXCL9*_*CD8A*_*XPA*_*ERCC2*_*ERCC5*	0.77	5
*CXCL9*_*CD274*_*XPA*_*ERCC4*_*ERCC1*	0.77	5
*CD8A*_*CD274*_*DDB2*_*ERCC1*_cluster 1 XP score	0.77	5
*CD274*_*XPA*_*XPC*_*ERCC4*_*ERCC1*	0.77	5
*CD274*_*DDB2*_*ERCC4*_*ERCC1*_cluster 1 XP score	0.77	5
*CD8A*_*ERCC2*_*ERCC1*_cluster 1 XP score	0.76	4
*ERCC3*_*ERCC4*_*ERCC1*_cluster 1 XP score	0.76	4
*XPC*_*ERCC4*_*ERCC1*	0.75	3
TIS score_*XPA*_*ERCC2*_cluster 1 XP score	0.75	4
*CXCL9*_*CD8A*_*XPC*_cluster 1 XP score	0.75	4
*CXCL9*_*CD274*_*ERCC1*_cluster 2 XP score	0.75	4
TIS score_*CXCL13*_*CD8A*_*ERCC4*_*ERCC1*	0.75	5
TIS score_*CD8A*_*CD274*_*ERCC1*_cluster 1 XP score	0.75	5
TIS score_*CD8A*_*CD274*_cluster 1 XP score_cluster 2 XP score	0.75	5
TIS score_*CD8A*_*ERCC3*_*ERCC4*_cluster 1 XP score	0.75	5
TIS score_*CD274*_*ERCC2*_*ERCC4*_cluster 1 XP score	0.75	5

Our study has several limitations. First, we restricted our analysis to melanoma, and the samples sizes of the two clinical cohorts that we analyzed were small, limiting our results’ comparability. For example, TIS score and genes indicative of immune infiltration, which, except for *CD8A*, performed poor as singular splitting parameters in our pooled data of two anti-PD-1 cohorts (11, 12), were significantly predictive in the study of Cristescu et al. (AUC of TIS score = 0.638) (7, 10). Second, due to the standardized pre-processing, the sample size of the two cohorts had to be further reduced. For the anti-PD-1 cohorts, we did not analyze survival data restricting our analysis to ICI response, which is not an appropriate measure of long-term treatment benefit. Third, 11 of 26 patients included from the cohort of Hugo et al. (11) and 26 of 49 patients in the cohort of Riaz et al. (12) were not treatment-naïve and had received prior MAPK inhibitor treatment or anti-CTLA-4 ICI, respectively, before sample acquisition, potentially influencing gene expression. Nevertheless, primary and acquired resistance constitutes a major problem in the systemic therapy of melanoma, suggesting the analysis of the XP gene clusters in additional patients with therapy failure (2, 3, 6, 81). Fourth, in the two clinical cohorts that we considered, mainly metastatic tissue was analyzed, and our analysis was made regardless of gender and age, which could have had an influence, especially on XP GEPs. Fifth, due to limited data, TMB and clonal TMB, which were the best performing predictive markers in the meta-analysis of Litchfield et al. (10), could not be assessed in our study. Likewise, we did also not assess the expression of genes in other DDR pathways.

Despite all these limitations, our analysis provides significant new findings that deserve attention: Firstly, XP genes are expressed in two heterogeneous clusters in melanoma. Secondly, these clusters correlate differentially with markers of a T cell-inflamed TME, and correlations depend to a certain degree on melanoma tissue origin (primary vs metastatic), age, and gender. Thirdly, a higher *ERCC3* expression could be associated with a better prognosis in melanoma, especially in younger patients. Lastly, the differentiated consideration of XP gene expression in the TME and its combination with established ICI predictive biomarkers could be useful in predicting anti-PD-1 ICI response in melanoma and should be explored by further studies.

## Data Availability Statement

The original contributions presented in the study are included in the article/**Supplementary Material**. Further inquiries can be directed to the corresponding author.

## Author Contributions

SF and AT designed this study, conducted the analysis, and wrote the article. MH, SE, OW and GF provided conceptual advice and critically reviewed the article. All authors contributed to the article and approved the submitted version.

## Funding

AT is supported by the “Hiege Foundation—the German Skin Cancer Foundation” and the “Forschungsförderung der Universitätsmedizin Rostock (FORUN)”. Support for computing equipment was provided by the European Union (EFRE, “Europäischer Fonds für regionale Entwicklung”). The funders had no role in the study design, collection, analysis, interpretation of the data, writing of the manuscript, or the decision to submit the paper for publication.

## Conflict of Interest

The authors declare that the research was conducted in the absence of any commercial or financial relationships that could be construed as a potential conflict of interest.

## Publisher’s Note

All claims expressed in this article are solely those of the authors and do not necessarily represent those of their affiliated organizations, or those of the publisher, the editors and the reviewers. Any product that may be evaluated in this article, or claim that may be made by its manufacturer, is not guaranteed or endorsed by the publisher.

## References

[1] Robert C. A Decade of Immune-Checkpoint Inhibitors in Cancer Therapy. Nat Commun (2020) 11(1):3801. doi: 10.1038/s41467-020-17670-y 32732879 PMC7393098

[2] Moreira A Heinzerling L Bhardwaj N Friedlander P. Current Melanoma Treatments: Where Do We Stand? Cancers (Basel) (2021) 13(2):221. doi: 10.3390/cancers13020221 PMC7827568 33435389

[3] Schadendorf D van Akkooi ACJ Berking C Griewank KG Gutzmer R Hauschild A. Melanoma. Lancet (2018) 392(10151):971–84. doi: 10.1016/S0140-6736(18)31559-9 30238891

[4] Larkin J Chiarion-Sileni V Gonzalez R Grob JJ Rutkowski P Lao CD. Five-Year Survival With Combined Nivolumab and Ipilimumab in Advanced Melanoma. N Engl J Med (2019) 381(16):1535–46. doi: 10.1056/NEJMoa1910836 31562797

[5] Havel JJ Chowell D Chan TA. The Evolving Landscape of Biomarkers for Checkpoint Inhibitor Immunotherapy. Nat Rev Cancer (2019) 19(3):133–50. doi: 10.1038/s41568-019-0116-x PMC6705396 30755690

[6] Sharma P Siddiqui BA Anandhan S Yadav SS Subudhi SK Gao J. The Next Decade of Immune Checkpoint Therapy. Cancer Discov (2021) 11(4):838–57. doi: 10.1158/2159-8290.CD-20-1680 33811120

[7] Cristescu R Mogg R Ayers M Albright A Murphy E Yearley J. Pan-Tumor Genomic Biomarkers for PD-1 Checkpoint Blockade-Based Immunotherapy. Science (2018) 362(6411):eaar3593. doi: 10.1126/science.aar3593 30309915 PMC6718162

[8] Gnjatic S Bronte V Brunet LR Butler MO Disis ML Galon J. Identifying Baseline Immune-Related Biomarkers to Predict Clinical Outcome of Immunotherapy. J Immunother Cancer (2017) 5(1):44. doi: 10.1186/s40425-017-0243-4 28515944 PMC5432988

[9] Ayers M Lunceford J Nebozhyn M Murphy E Loboda A Kaufman DR. IFN-Gamma-Related mRNA Profile Predicts Clinical Response to PD-1 Blockade. J Clin Invest (2017) 127(8):2930–40. doi: 10.1172/JCI91190 PMC5531419 28650338

[10] Litchfield K Reading JL Puttick C Thakkar K Abbosh C Bentham R. Meta-Analysis of Tumor- and T Cell-Intrinsic Mechanisms of Sensitization to Checkpoint Inhibition. Cell (2021) 184(3):596–614.e14. doi: 10.1016/j.cell.2021.01.002 33508232 PMC7933824

[11] Hugo W Zaretsky JM Sun L Song C Moreno BH Hu-Lieskovan S. Genomic and Transcriptomic Features of Response to Anti-PD-1 Therapy in Metastatic Melanoma. Cell (2016) 165(1):35–44. doi: 10.1016/j.cell.2016.02.065 26997480 PMC4808437

[12] Riaz N Havel JJ Makarov V Desrichard A Urba WJ Sims JS. Tumor and Microenvironment Evolution During Immunotherapy With Nivolumab. Cell (2017) 171(4):934–49.e16. doi: 10.1016/j.cell.2017.09.028 29033130 PMC5685550

[13] Bever KM Le DT. DNA Repair Defects and Implications for Immunotherapy. J Clin Invest (2018) 128(10):4236–42. doi: 10.1172/JCI122010 PMC6159999 30272580

[14] Keenan TE Burke KP Van Allen EM. Genomic Correlates of Response to Immune Checkpoint Blockade. Nat Med (2019) 25(3):389–402. doi: 10.1038/s41591-019-0382-x 30842677 PMC6599710

[15] Mouw KW D'Andrea AD. DNA Repair Deficiency and Immunotherapy Response. J Clin Oncol (2018) 36(17):1710–3. doi: 10.1200/JCO.2018.78.2425 29683789

[16] Teo MY Seier K Ostrovnaya I Regazzi AM Kania BE Moran MM. Alterations in DNA Damage Response and Repair Genes as Potential Marker of Clinical Benefit From PD-1/PD-L1 Blockade in Advanced Urothelial Cancers. J Clin Onc ol (2018) 36(17):1685–94. doi: 10.1200/JCO.2017.75.7740 PMC6366295 29489427

[17] Germano G Lamba S Rospo G Barault L Magri A Maione F. Inactivation of DNA Repair Triggers Neoantigen Generation and Impairs Tumour Growth. Nature (2017) 552(7683):116–20. doi: 10.1038/nature24673 29186113

[18] Le DT Durham JN Smith KN Wang H Bartlett BR Aulakh LK. Mismatch Repair Deficiency Predicts Response of Solid Tumors to PD-1 Blockade. Science (2017) 357(6349):409–13. doi: 10.1126/science.aan6733 PMC5576142 28596308

[19] Le DT Uram JN Wang H Bartlett BR Kemberling H Eyring AD. PD-1 Blockade in Tumors With Mismatch-Repair Deficiency. N Engl J Med (2015) 372(26):2509–20. doi: 10.1056/NEJMoa1500596 PMC4481136 26028255

[20] Schumacher TN Schreiber RD. Neoantigens in Cancer Immunotherapy. Science (2015) 348(6230):69–74. doi: 10.1126/science.aaa4971 25838375

[21] Gladbach YS Wiegele L Hamed M Merkenschlager AM Fuellen G Junghanss C. Unraveling the Heterogeneous Mutational Signature of Spontaneously Developing Tumors in MLH1(-/-) Mice. Cancers (Basel) (2019) 11(10):1485. doi: 10.3390/cancers11101485 PMC6827043 31581674

[22] Jeggo PA Pearl LH Carr AM. DNA Repair, Genome Stability and Cancer: A Historical Perspective. Nat Rev Cancer (2016) 16(1):35–42. doi: 10.1038/nrc.2015.4 26667849

[23] Patterson-Fortin J D'Andrea AD. Exploiting the Microhomology-Mediated End-Joining Pathway in Cancer Therapy. Cancer Res (2020) 80(21):4593–600. doi: 10.1158/0008-5472.Can-20-1672 PMC7641946 32651257

[24] Sfeir A Symington LS. Microhomology-Mediated End Joining: A Back-Up Survival Mechanism or Dedicated Pathway? Trends Biochem Sci (2015) 40(11):701–14. doi: 10.1016/j.tibs.2015.08.006 PMC4638128 26439531

[25] Stewart RA Pilie PG Yap TA. Development of PARP and Immune-Checkpoint Inhibitor Combinations. Cancer Res (2018) 78(24):6717–25. doi: 10.1158/0008-5472.CAN-18-2652 30498083

[26] Permata TBM Hagiwara Y Sato H Yasuhara T Oike T Gondhowiardjo S. Base Excision Repair Regulates PD-L1 Expression in Cancer Cells. Oncogene (2019) 38(23):4452–66. doi: 10.1038/s41388-019-0733-6 30755733

[27] Sato H Niimi A Yasuhara T Permata TBM Hagiwara Y Isono M. DNA Double-Strand Break Repair Pathway Regulates PD-L1 Expression in Cancer Cells. Nat Commun (2017) 8(1):1751. doi: 10.1038/s41467-017-01883-9 29170499 PMC5701012

[28] Garcia-Diaz A Shin DS Moreno BH Saco J Escuin-Ordinas H Rodriguez GA. Interferon Receptor Signaling Pathways Regulating PD-L1 and PD-L2 Expression. Cell Rep (2017) 19(6):1189–201. doi: 10.1016/j.celrep.2017.04.031 PMC6420824 28494868

[29] Parkes EE Walker SM Taggart LE McCabe N Knight LA Wilkinson R. Activation of STING-Dependent Innate Immune Signaling By S-Phase-Specific DNA Damage in Breast Cancer. J Natl Cancer Inst (2016) 109(1):djw199. doi: 10.1093/jnci/djw199 PMC5441301 27707838

[30] Trujillo JA Sweis RF Bao R Luke JJ. T Cell-Inflamed Versus Non-T Cell-Inflamed Tumors: A Conceptual Framework for Cancer Immunotherapy Drug Development and Combination Therapy Selection. Cancer Immunol Res (2018) 6(9):990–1000. doi: 10.1158/2326-6066.CIR-18-0277 30181337 PMC6145135

[31] Ribas A. Adaptive Immune Resistance: How Cancer Protects From Immune Attack. Cancer Discov (2015) 5(9):915–9. doi: 10.1158/2159-8290.CD-15-0563 PMC4560619 26272491

[32] Mouw KW Goldberg MS Konstantinopoulos PA D'Andrea AD. DNA Damage and Repair Biomarkers of Immunotherapy Response. Cancer Discov (2017) 7(7):675–93. doi: 10.1158/2159-8290.Cd-17-0226 PMC5659200 28630051

[33] Chambon F Osdoit S Bagny K Moro A Nguyen J Reguerre Y. Dramatic Response to Nivolumab in Xeroderma Pigmentosum Skin Tumor. Pediatr Blood Cancer (2018) 65(2):e26837. doi: 10.1002/pbc.26837 28988442

[34] Deinlein T Lax SF Schwarz T Giuffrida R Schmid-Zalaudek K Zalaudek I. Rapid Response of Metastatic Cutaneous Squamous Cell Carcinoma to Pembrolizumab in a Patient With Xeroderma Pigmentosum: Case Report and Review of the Literature. Eur J Cancer (2017) 83:99–102. doi: 10.1016/j.ejca.2017.06.022 28734147

[35] Hauschild A Eichstaedt J Mobus L Kahler K Weichenthal M Schwarz T. Regression of Melanoma Metastases and Multiple Non-Melanoma Skin Cancers in Xeroderma Pigmentosum by the PD1-Antibody Pembrolizumab. Eur J Cancer (2017) 77:84–7. doi: 10.1016/j.ejca.2017.02.026 28365530

[36] Salomon G Maza A Boulinguez S Paul C Lamant L Tournier E. Efficacy of Anti-Programmed Cell Death-1 Immunotherapy for Skin Carcinomas and Melanoma Metastases in a Patient With Xeroderma Pigmentosum. Br J Dermatol (2018) 178(5):1199–203. doi: 10.1111/bjd.16270 29274233

[37] Steineck A Krumm N Sarthy JF Pritchard CC Chapman T Stacey AW. Response to Pembrolizumab in a Patient With Xeroderma Pigmentosum and Advanced Squamous Cell Carcinoma. JCO Precis Oncol (2019) 3:PO.19.00028. doi: 10.1200/PO.19.00028 32923855 PMC7446378

[38] Kraemer KH DiGiovanna JJ. Xeroderma Pigmentosum. In: Adam MP Ardinger HH Pagon RA, editors. GeneReviews® [Internet]. Seattle (WA): University of Washington, Seattle 1993–2021 (2003). Available at: https://www.ncbi.nlm.nih.gov/books/NBK1397/ 20301571

[39] Marteijn JA Lans H Vermeulen W Hoeijmakers JH. Understanding Nucleotide Excision Repair and Its Roles in Cancer and Ageing. Nat Rev Mol Cell Biol (2014) 15(7):465–81. doi: 10.1038/nrm3822 24954209

[40] Bradford PT Goldstein AM Tamura D Khan SG Ueda T Boyle J. Cancer and Neurologic Degeneration in Xeroderma Pigmentosum: Long Term Follow-Up Characterises the Role of DNA Repair. J Med Genet (2011) 48(3):168–76. doi: 10.1136/jmg.2010.083022 PMC3235003 21097776

[41] Hsiehchen D Hsieh A Samstein RM Lu T Beg MS Gerber DE. DNA Repair Gene Mutations as Predictors of Immune Checkpoint Inhibitor Response Beyond Tumor Mutation Burden. Cell Rep Med (2020) 1(3):100034. doi: 10.1016/j.xcrm.2020.100034 32676589 PMC7365618

[42] Samstein RM Krishna C Ma X Pei X Lee KW Makarov V. Mutations in BRCA1 and BRCA2 Differentially Affect the Tumor Microenvironment and Response to Checkpoint Blockade Immunotherapy. Nat Cancer (2021) 1(12):1188–203. doi: 10.1038/s43018-020-00139-8 PMC8023400 33834176

[43] Genomic Classification of Cutaneous Melanoma. Cell (2015) 161(7):1681–96. doi: 10.1016/j.cell.2015.05.044 PMC4580370 26091043

[44] Oldenhuis CN Oosting SF Gietema JA de Vries EG. Prognostic Versus Predictive Value of Biomarkers in Oncology. Eur J Cancer (2008) 44(7):946–53. doi: 10.1016/j.ejca.2008.03.006 18396036

[45] Ayers M Loboda A Lunceford J Mcclanahan T Murphy E Nebozhyn M. WO2016094377 (2016). 2016-06-16. Available at: https://p ubchem.n cbi.nlm.nih.gov/patent/WO-2016094377- A1

[46] Team RC. R: A Language and Environment for Statistical Computing Vienna, Austria. Vienna, Austria: R Foundation for Statistical Computing (2019). Available at: https://www.R-project.org/.

[47] Mayakonda A Lin DC Assenov Y Plass C Koeffler HP. Maftools: Efficient and Comprehensive Analysis of Somatic Variants in Cancer. Genome Res (2018) 28(11):1747–56. doi: 10.1101/gr.239244.118 PMC6211645 30341162

[48] Revelle W. Psych: Procedures for Psychological, Psychometric, and Personality Research (2021). Available at: https://CRAN.R-project.org/package=psych.

[49] Gu Z Eils R Schlesner M. Complex Heatmaps Reveal Patterns and Correlations in Multidimensional Genomic Data. Bioinformatics (2016) 32(18):2847–9. doi: 10.1093/bioinformatics/btw313 27207943

[50] Therneau TM Grambsch PM. Modeling Survival Data: Extending the Cox Model. New York: Springer Nature (2000).

[51] Therneau TM. A Package for Survival Analysis in R (2021). Available at: https://CRAN.R-project.org/package=survival.

[52] Kassambara A Kosinski M Biecek P. Survminer: Drawing Survival Curves Using 'Ggplot2' (2021). Available at: https://cran.r-project.org/web/packages/survminer.

[53] Thiele C. Cutpointr: Determine and Evaluate Optimal Cutpoints in Binary Classification Tasks (2021). Available at: https://cran.r-project.org/web/packages/cutpointr.

[54] Thiele C Hirschfeld G. Cutpointr: Improved Estimation and Validation of Optimal Cutpoints in R. J Stat Softw (2021) 98(11):1 – 27. doi: 10.18637/jss.v098.i11

[55] Chen T He T Benesty M Khotilovich V Tang Y Cho H. Xgboost: Extreme Gradient Boosting (2021). Available at: https://CRAN.R-project.org/package=xgboost.

[56] Kuhn M. Caret: Classification and Regression Training (2020). Available at: https://CRAN.R-project.org/package=caret.

[57] Hoadley KA Yau C Hinoue T Wolf DM Lazar AJ Drill E. Cell-Of-Origin Patterns Dominate the Molecular Classification of 10,000 Tumors From 33 Types of Cancer. Cell (2018) 173(2):291–304.e6. doi: 10.1016/j.cell.2018.03.022 29625048 PMC5957518

[58] Vidman L Kallberg D Ryden P. Cluster Analysis on High Dimensional RNA-Seq Data With Applications to Cancer Research - An Evaluation Study. PloS One (2019) 14(12):e0219102. doi: 10.1371/journal.pone.0219102 31805048 PMC6894875

[59] Vitale I Shema E Loi S Galluzzi L. Intratumoral Heterogeneity in Cancer Progression and Response to Immunotherapy. Nat Med (2021) 27(2):212–24. doi: 10.1038/s41591-021-01233-9 33574607

[60] Scharer OD. Nucleotide Excision Repair in Eukaryotes. Cold Spring Harb Perspect Biol (2013) 5(10):a012609. doi: 10.1101/cshperspect.a012609 24086042 PMC3783044

[61] Orelli B McClendon TB Tsodikov OV Ellenberger T Niedernhofer LJ Scharer OD. The XPA-Binding Domain of ERCC1 Is Required for Nucleotide Excision Repair But Not Other DNA Repair Pathways. J Biol Chem (2010) 285(6):3705–12. doi: 10.1074/jbc.M109.067538 PMC2823511 19940136

[62] Tsodikov OV Ivanov D Orelli B Staresincic L Shoshani I Oberman R. Structural Basis for the Recruitment of ERCC1-XPF to Nucleotide Excision Repair Complexes by XPA. EMBO J (2007) 26(22):4768–76. doi: 10.1038/sj.emboj.7601894 PMC2080803 17948053

[63] McNeil EM Melton DW. DNA Repair Endonuclease ERCC1-XPF as a Novel Therapeutic Target to Overcome Chemoresistance in Cancer Therapy. Nucleic Acids Res (2012) 40(20):9990–10004. doi: 10.1093/nar/gks818 22941649 PMC3488251

[64] Deloia JA Bhagwat NR Darcy KM Strange M Tian C Nuttall K. Comparison of ERCC1/XPF Genetic Variation, mRNA and Protein Levels in Women With Advanced Stage Ovarian Cancer Treated With Intraperitoneal Platinum. Gynecol Oncol (2012) 126(3):448–54. doi: 10.1016/j.ygyno.2012.05.006 PMC3518863 22609620

[65] Biertumpfel C Zhao Y Kondo Y Ramon-Maiques S Gregory M Lee JY. Structure and Mechanism of Human DNA Polymerase Eta. Nature (2010) 465(7301):1044–8. doi: 10.1038/nature09196 PMC2899710 20577208

[66] Boonstra A van Oudenaren A Baert M van Steeg H Leenen PJ van der Horst GT. Differential Ultraviolet-B-Induced Immunomodulation in XPA, XPC, and CSB DNA Repair-Deficient Mice. J Invest Dermatol (2001) 117(1):141–6. doi: 10.1046/j.0022-202x.2001.01390.x 11442761

[67] Fontes FL Pinheiro DM Oliveira AH Oliveira RK Lajus TB Agnez-Lima LF. Role of DNA Repair in Host Immune Response and Inflammation. Mutat Res Rev Mutat Res (2015) 763:246–57. doi: 10.1016/j.mrrev.2014.11.004 25795123

[68] Altman BJ Hsieh AL Sengupta A Krishnanaiah SY Stine ZE Walton ZE. MYC Disrupts the Circadian Clock and Metabolism in Cancer Cells. Cell Metab (2015) 22(6):1009–19. doi: 10.1016/j.cmet.2015.09.003 PMC4818967 26387865

[69] Kang T-H. Circadian Rhythm of NER and ATR Pathways. Biomolecules (2021) 11(5):715. doi: 10.3390/biom11050715 34064641 PMC8150605

[70] Luo H Ma C Shao J Cao J. Prognostic Implications of Novel Ten-Gene Signature in Uveal Melanoma. Front Oncol (2020) 10:567512(2154). doi: 10.3389/fonc.2020.567512 33194647 PMC7661968

[71] Tong X Qu X Wang M. A Four-Gene-Based Prognostic Model Predicts Overall Survival in Patients With Cutaneous Melanoma. Front Oncol (2021) 11:639874(400). doi: 10.3389/fonc.2021.639874 33842346 PMC8024561

[72] Zhang JA Zhou XY Huang D Luan C Gu H Ju M. Development of an Immune-Related Gene Signature for Prognosis in Melanoma. Front Oncol (2020) 10:602555(3280). doi: 10.3389/fonc.2020.602555 33585219 PMC7874014

[73] Danaher P Warren S Lu R Samayoa J Sullivan A Pekker I. Pan-Cancer Adaptive Immune Resistance as Defined by the Tumor Inflammation Signature (TIS): Results From The Cancer Genome Atlas (TCGA). J Immunother Cancer (2018) 6(1):63. doi: 10.1186/s40425-018-0367-1 29929551 PMC6013904

[74] McNamara MG Jacobs T Lamarca A Hubner RA Valle JW Amir E. Impact of High Tumor Mutational Burden in Solid Tumors and Challenges for Biomarker Application. Cancer Treat Rev (2020) 89:102084. doi: 10.1016/j.ctrv.2020.102084 32738738

[75] Weiss SA Han J Darvishian F Tchack J Han SW Malecek K. Impact of Aging on Host Immune Response and Survival in Melanoma: An Analysis of 3 Patient Cohorts. J Transl Med (2016) 14(1):299. doi: 10.1186/s12967-016-1026-2 27760559 PMC5070187

[76] Xiong J Bing Z Guo S. Observed Survival Interval: A Supplement to TCGA Pan-Cancer Clinical Data Resource. Cancers (Basel) (2019) 11(3):280. doi: 10.3390/cancers11030280 PMC6468755 30813652

[77] Wu HX Wang ZX Zhao Q Chen DL He MM Yang LP. Tumor Mutational and Indel Burden: A Systematic Pan-Cancer Evaluation as Prognostic Biomarkers. Ann Transl Med (2019) 7(22):640. doi: 10.21037/atm.2019.10.116 31930041 PMC6944566

[78] Si Z Hu H. Identification of CXCL13 as an Immune-Related Biomarker Associated With Tumorigenesis and Prognosis in Cutaneous Melanoma Patients. Med Sci Monit (2021) 27:e932052. doi: 10.12659/msm.932052 34247183 PMC8280950

[79] Zhou X Peng M He Y Peng J Zhang X Wang C. CXC Chemokines as Therapeutic Targets and Prognostic Biomarkers in Skin Cutaneous Melanoma Microenvironment. Front Oncol (2021) 11:619003. doi: 10.3389/fonc.2021.619003 33767987 PMC7985846

[80] Spranger S Luke JJ Bao R Zha Y Hernandez KM Li Y. Density of Immunogenic Antigens Does Not Explain the Presence or Absence of the T-Cell-Inflamed Tumor Microenvironment in Melanoma. Proc Natl Acad Sci USA (2016) 113(48):E7759–68. doi: 10.1073/pnas.1609376113 PMC5137753 27837020

[81] Bai R Chen N Li L Du N Bai L Lv Z. Mechanisms of Cancer Resistance to Immunotherapy. Front Oncol (2020) 10:1290. doi: 10.3389/fonc.2020.01290 32850400 PMC7425302

