# Supplementary material for: The Prognostic and Predictive Role of Xeroderma Pigmentosum Gene Expression in Melanoma
Source: Front Oncol. 2022 Jan 31;12:810058. doi: 10.3389/fonc.2022.810058 (PMC8841870; doi:10.3389/fonc.2022.810058)
Supplement: Supplementary file 1 [file DataSheet_1.docx]

Supplementary Material

# Supplementary Tables

## Supplementary Table S1:

The clinical characteristics and the range of computed scores in the analyzed melanoma cohorts.

*Hugo et al. provided only the M category. Because 1 sample was classified M0, for this sample a stadium assignation could not be made.

** 58 was selected based on the median age in the TCGA-SKCM cohort

| **Cohorts** | | **TCGA-SKCM** | | | **Hugo et al.** | **Riaz et al.** |
| --- | --- | --- | --- | --- | --- | --- |
|  |  |  | Older  patients  (≥ 58**) | Younger  patients  (< 58**) |  |  |
| Original size, n | | 464 |  |  | 28 | 110 |
| Pre-processed samples, n (%) | | 445 (100%) | 233 (100 %) | 212 (100%) | 26 | 49 |
| **Clinical Information** | | | | | | |
| Vital status at last follow-up | Alive | 230  (51,7 %) | 122  (52,3%) | 108  (50,9%) | 15  (57,7%) | Not reported |
|  | Dead  Median follow-up (days) | 215 (48,3%)  669.50 | 111  (47,7%)  355 | 104  (49,1%)  991 | 11  (42,3%) |  |
| Overall survival (days) | Median | 1103 | 955 | 1433.5 | 443.5 | Not reported |
|  | Min - Max | 79- 10870 | 79-10346 | 151-10870 | 54-1060 |  |
| Gender | Female | 276 | 141 | 135 | 18 | Not reported |
|  | Male | 169 | 92 | 77 | 8 |  |
| Disease stage | Stage 0 | 6 | 2 | 4 |  | Not reported |
|  | Stage I | 74 | 28 | 46 |  |  |
|  | Stage II | 135 | 99 | 36 |  |  |
|  | Stage III | 166 | 78 | 88 |  |  |
|  | Stage IV | 21 | 12 | 9 | 25 |  |
|  | Not reported | 43 | 14 | 29 | 1* |  |
| Age (years) | Median | 58 | 70 | 47 | 61.50 | Not reported |
|  | Min- Max | 15 - 87 | 58 - 87 | 15 - 57 | 27 - 84 |  |
| Computed scores | | | | | | |
| TIS score | Median | 3.573 | 3.4567 | 3.712 | 4.16 | 3.740 |
|  | Min-Max | -Inf –  8.105 | -0.8179-  8.1049 | -Inf –  7.874 | 0.02705-  7.17050 | -Inf-  6.386 |
| XP Cluster 1 score | Median | 4.29 | 4.155 | 4.439 | 7.368 | 2.91 |
|  | Min-Max | -3.95-  6.859 | -3.95-  6.696 | -2.906-  6.859 | 3.280-  9.626 | -0.3282-  4.7259 |
| XP Cluster 2 score | Median | 19.08 | 19.15 | 18.76 | 23.13 | 19.03 |
|  | Min-Max | 10.49-  24.62 | 12.26-  23.36 | 10.49-  24.62 | 20.58-  28.92 | 13.11-  25.20 |
| Treatment response | Responder  (CR, PR) | Not reported | Not reported | Not reported | 14 | 10 |
|  | Non-responder (SD, PD) | Not reported | Not reported | Not reported | 12 | 39 |

## Supplementary Table S2:

Executed different clustering methods and distance metrics used in hierarchical clustering

| **Distance metrics** | **Clustering methods** |
| --- | --- |
| Ward.D | Euclidean |
| Ward.D2 | Maximum |
| Single | Manhatten |
| Complete | Canberra |
| Average | Binary |
| Mcquitty | Minkowski |
| Median |  |
| Centroid |  |

## Supplementary Table S3:

Signature genes of the 18-gene score with their predefined weight of Ayers et al, which were used to compute the TIS score.

| **Gene in TIS score** | **Predefined Weights** |
| --- | --- |
| *CD27* | 0.072293 |
| *CD8A* | 0.031021 |
| *PDCD1LG2* | 0.003734 |
| *LAG3* | 0.123895 |
| *CD274* | 0.042853 |
| *CXCR6* | 0.004313 |
| *CMKLR1* | 0.151253 |
| *NKG7* | 0.075524 |
| *CCL5* | 0.008346 |
| *PSMB10* | 0.032999 |
| *IDO1* | 0.060679 |
| *CXCL9* | 0.074135 |
| *HLA.DQA1* | 0.020091 |
| *CD276* | -0.0239 |
| *STAT1* | 0.250229 |
| *HLA.DRB1* | 0.058806 |
| *HLA.E* | 0.07175 |
| *TIGIT* | 0.084767 |

## Supplementary Table S4:

XP gene expression range in the different stratified patient subcohorts with associated clusters.

| **Dataset** | ***XPA*** | ***ERCC4*** | ***ERCC5*** | ***ERCC3*** | ***XPC*** | ***ERCC2*** | ***DDB2*** | ***POLH*** | ***ERCC1*** |
| --- | --- | --- | --- | --- | --- | --- | --- | --- | --- |
| Cluster Association | 1 | 1 | 1 | 2 | 2 | 2 | 2 | 2 | 2 |
| All patients (n=445) expression  range | 2.15: -0.69 - 2.54 | 0.44:  -3.02 – 0.76 | 1.58:  -1.39 - 1.92 | 3.18: 0.94 - 3.43 | 3.4: 0.66 - 3.79 | 2.7: 0.33 - 3.11 | 2.87: -0.11 - 3.43 | 2.97: 0.81 - 3.48 | 3.85: 2.01 - 4.19 |
| Primary samples (n=96) expression range | 2.1: -0.21 - 2.52 | 0.13:  -3.02 - 0.58 | 1.53:  -1.39 - 1.9 | 3.15: 0.94 - 3.34 | 3.29: 0.66 - 3.73 | 2.65: 0.33 - 3.04 | 3.1: 0.63 - 3.66 | 2.88: 0.92 - 3.4 | 3.92: 2.64 - 4.22 |
| Metastatic (n=349) expression range | 2.18: -0.69 - 2.56 | 0.47:  -2.61 - 0.82 | 1.6:  -0.78 - 1.94 | 3.19: 1.65 - 3.44 | 3.43: 1.42 - 3.82 | 2.71: 1.16 - 3.12 | 2.8:  -0.11 - 3.39 | 3.05: 0.81 - 3.49 | 3.84: 2.01 - 4.19 |
| Younger patients (n=212) expression range | 2.18: -0.69 - 2.55 | 0.45:  -2.11 - 0.76 | 1.64:  -0.78 - 1.97 | 3.16: 0.94 - 3.38 | 3.45: 0.66 - 3.78 | 2.63: 0.33 - 3.06 | 2.89: 0.55 - 3.41 | 2.89: 0.81 - 3.32 | 3.83: 2.01 - 4.19 |
| Older patients (n=233) expression range | 2.09: -0.29 - 2.53 | 0.4:  -3.02 - 0.76 | 1.52:  -1.39 - 1.86 | 3.21: 1.73 - 3.48 | 3.33: 0.72 - 3.83 | 2.79: 1.12 - 3.12 | 2.84: -0.11 - 3.44 | 3.06: 1.05 - 3.52 | 3.86: 2.57 - 4.18 |
| Female (n=169)  expression range | 2.19: 0.16 - 2.61 | 0.46:  -2.11 - 0.8 | 1.59:  -0.54 - 1.92 | 3.19: 0.94 - 3.44 | 3.45: 0.66 - 3.84 | 2.64: 0.33 - 3.07 | 2.9:  -0.11 - 3.47 | 3.01: 0.92 - 3.46 | 3.8: 2.01 - 4.13 |
| Male (n=276)  expression range | 2.09: -0.69 - 2.5 | 0.38:  -3.02 - 0.71 | 1.56:  -1.39 - 1.92 | 3.17: 1.65 - 3.41 | 3.35: 0.72 - 3.71 | 2.8: 1.28 - 3.19 | 2.83: 0.57 - 3.35 | 2.94: 0.81 - 3.48 | 3.95: 2.57 - 4.24 |

## Supplementary Table S5:

TCGA-SKCM list of drugs applied after diagnosis and recording by the NIH.

| **Drug Name** | **Number of patients treated (n)** | **ICI drugs** |
| --- | --- | --- |
| 2b3-101 | 1 |  |
| actinomycin d | 1 |  |
| actinomycin-d | 1 |  |
| alkeran | 1 |  |
| axitinib | 1 |  |
| braf inhibitor | 1 |  |
| cancer vax | 2 |  |
| carboplatin | 2 |  |
| carmustine | 1 |  |
| cisplatin | 6 |  |
| cyclophosphamide | 1 |  |
| dabrafenib | 2 |  |
| dacabarzine | 1 |  |
| dacarbazine | 18 |  |
| diphencyprone | 1 |  |
| dtic | 2 |  |
| fareston | 1 |  |
| fotemustine | 3 |  |
| gm-csf | 1 |  |
| gp100 | 1 |  |
| il-18 | 1 |  |
| interferon | 19 |  |
| interferon alfa | 1 |  |
| interferon alfa-2b | 1 |  |
| interferon alpha | 5 |  |
| interferon-alfa | 1 |  |
| interleukin-2 | 2 |  |
| ipilimumab | 14 | CTLA-4 ICI |
| lomustine | 1 |  |
| lupron | 1 |  |
| mage a3 | 1 |  |
| mel-44 | 2 |  |
| melphalan | 2 |  |
| nivolumab | 1 | Anti- PD1 ICI |
| paclitaxel | 2 |  |
| patrin | 1 |  |
| pembrolizumab | 1 | Anti- PD1 ICI |
| pi88 | 1 |  |
| pnu-159548 | 1 |  |
| pv-10 | 1 |  |
| recmage- a3 | 1 |  |
| sylatron | 2 |  |
| talimogene laherparepvec (t-vec) | 1 |  |
| tamoxifen | 2 |  |
| taxol | 1 |  |
| temodal | 1 |  |
| temodar | 2 |  |
| temozolomide | 1 |  |
| vemurafenib | 6 |  |
| vmcl | 1 |  |
| yervoy | 2 | CTLA-4 ICI |

**Supplementary** **Table S6:**

List of AUCs based on ROC curves constructed for each parameter using the Youden index.

| **Parameter** | **AUC** | **Youden index associated cutoff points** |
| --- | --- | --- |
| *CD8A* | 0.627 | 2.892 |
| *CD274* | 0.533 | 2.862 |
| XP cluster 1 score | 0.654 | 7.311 |
| XP cluster 2 score | 0.632 | 21.738 |
| *CXCL9* | 0.570 | 4.816 |
| *CXCL13* | 0.613 | 2.703 |
| *DDB2* | 0.570 | 3.390 |
| *ERCC1* | 0.621 | 4.835 |
| *ERCC2* | 0.622 | 2.794 |
| *ERCC3* | 0.600 | 4.243 |
| *ERCC4* | 0.592 | 1.372 |
| *ERCC5* | 0.660 | 2.862 |
| TIS score | 0.586 | 3.632 |
| *POLH* | 0.545 | 2.685 |
| *XPA* | 0.532 | 3.179 |
| *XPC* | 0.587 | 4.094 |

**Supplementary** **Table S7**:

Top 25 performed classification models of parameter pairs utilizing the XgBoost method. The corresponding AUC values are retrieved from the test set partition (n=17).

| **Parameter for predictive model** | **AUC** |
| --- | --- |
| *CD274*_*ERCC4* | 0.70 |
| *ERCC2*_*ERCC5* | 0.69 |
| *CXCL13*_*ERCC5* | 0.68 |
| *CD274*_*ERCC5* | 0.66 |
| *XPA*_XP cluster 2 score | 0.66 |
| *ERCC2*_XP cluster 1 score | 0.65 |
| *ERCC4*_*POLH* | 0.65 |
| *ERCC5*_*ERCC1* | 0.64 |
| *ERCC5*_XP cluster 1 score | 0.64 |
| *CD274*_*ERCC1* | 0.63 |
| *CD274*_XP cluster 1 score | 0.63 |
| *ERCC4*_*ERCC5* | 0.63 |
| *ERCC1*_XP cluster 2 score | 0.63 |
| *CD8A*_XP cluster 1 score | 0.62 |
| *XPC*_XP cluster 2 score | 0.62 |
| *ERCC4*_XP cluster 1 score | 0.62 |
| *POLH*_XP cluster 2 score | 0.62 |
| *ERCC4*_*ERCC1* | 0.61 |
| *ERCC5*_XP cluster 2 score | 0.61 |
| *CXCL13*_*CD274* | 0.60 |
| *CXCL13*_XP cluster 1 score | 0.60 |
| *XPC*_*ERCC2* | 0.60 |
| *CD274*_*ERCC2* | 0.58 |
| *XPC*_*ERCC4* | 0.58 |
| *ERCC4*_XP cluster 2 score | 0.58 |

**Supplementary** **Table S8**:

Top 25 classification models, using parameter triplets for response prediction with XgBoost method. The corresponding AUC values were calculated on the test partition of the pooled response datasets (n=17).

| **Parameter for predictive model** | **AUC** |
| --- | --- |
| *ERCC3*_*XPC*_*ERCC4* | 0.80 |
| *XPC*_*ERCC4*_*ERCC1* | 0.75 |
| *ERCC4*_*ERCC1*_XP cluster 1 score | 0.73 |
| *XPA*_*ERCC2*_XP cluster 1 score | 0.72 |
| *ERCC2*_*ERCC4*_*ERCC1* | 0.72 |
| *CXCL13*_*CD274*_*ERCC5* | 0.70 |
| *CXCL9*_*CD274*_XP cluster 1 score | 0.70 |
| *CD274*_*POLH*_*ERCC1* | 0.70 |
| *CD274*_*ERCC1*_XP cluster 1 score | 0.70 |
| *ERCC2*_XP cluster 1 score_XP cluster 2 score | 0.70 |
| *CXCL13*_*ERCC4*_XP cluster 1 score | 0.68 |
| *CXCL13*_XP cluster 1 score_XP cluster 2 score | 0.68 |
| *CXCL9*_*ERCC2*_*ERCC4* | 0.68 |
| *CD8A*_*ERCC1*_XP cluster 1 score | 0.68 |
| *CD8A*_*ERCC1*_XP cluster 2 score | 0.68 |
| *CD274*_*XPC*_*ERCC5* | 0.68 |
| *CD274*_*ERCC2*_XP cluster 1 score | 0.68 |
| *XPC*_*ERCC2*_XP cluster 2 score | 0.68 |
| *DDB2*_*ERCC4*_XP cluster 1 score | 0.68 |
| *CD8A*_*XPC*_XP cluster 1 score | 0.67 |
| *CD8A*_*ERCC4*_XP cluster 2 score | 0.67 |
| *CD8A*_XP cluster 1 score_XP cluster 2 score | 0.67 |
| *CD274*_XP cluster 1 score_XP cluster 2 score | 0.67 |
| *XPA*_*ERCC4*_*ERCC1* | 0.67 |
| *ERCC3*_*ERCC4*_XP cluster 1 score | 0.67 |

## Supplementary Table S9:

Top 25 classification models constructed out of four parameters for response using the XgBoost method. The specification for top values is based on the given AUC values of the test set partition of the patients (n=17).

| **Parameter for predictive model** | **AUC** |
| --- | --- |
| *CD274*_*XPA*_*ERCC2*_*ERCC4* | 0.85 |
| TIS score_*ERCC2*_*ERCC1*_XP cluster 1 score | 0.78 |
| *CXCL9*_*ERCC3*_*ERCC4*_XP cluster 1 score | 0.77 |
| *CD8A*_*ERCC2*_*ERCC1*_XP cluster 1 score | 0.76 |
| *ERCC3*_*ERCC4*_*ERCC1*_XP cluster 1 score | 0.76 |
| TIS score_*XPA*_*ERCC2*_XP cluster 1 score | 0.75 |
| *CXCL9*_*CD8A*_*XPC*_XP cluster 1 score | 0.75 |
| *CXCL9*_*CD274*_*ERCC1*_XP cluster 2 score | 0.75 |
| *CXCL13*_*ERCC2*_*ERCC4*_*ERCC5* | 0.73 |
| *CXCL9*_*CD274*_*XPC*_*ERCC1* | 0.73 |
| *XPC*_*DDB2*_*ERCC4*_XP cluster 2 score | 0.73 |
| TIS score_*CD8A*_*ERCC5*_XP cluster 1 score | 0.72 |
| TIS score_*CD274*_*ERCC5*_*ERCC1* | 0.72 |
| TIS score_*ERCC3*_*ERCC4*_XP cluster 1 score | 0.72 |
| *CXCL13*_*CXCL9*_*CD274*_XP cluster 1 score | 0.72 |
| *CXCL13*_*CD8A*_*XPC*_XP cluster 1 score | 0.72 |
| *CD8A*_*ERCC1*_XP cluster 1 score_XP cluster 2 score | 0.72 |
| *CD274*_*XPA*_*ERCC4*_XP cluster 1 score | 0.72 |
| *CD274*_*ERCC3*_*ERCC1*_XP cluster 1 score | 0.72 |
| *POLH*_*ERCC1*_XP cluster 1 score_XP cluster 2 score | 0.72 |
| TIS score_*CXCL9*_*XPC*_*ERCC4* | 0.70 |
| TIS score_*CD274*_*XPA*_*ERCC5* | 0.70 |
| *CXCL13*_*CXCL9*_*CD274*_*ERCC1* | 0.70 |
| *CXCL13*_*CXCL9*_*ERCC2*_*ERCC5* | 0.70 |
| *CXCL13*_*CD274*_*ERCC5*_XP cluster 2 score | 0.70 |

## Supplementary Table S10:

Best 25 models for response using five parameters for classification with XgBoost method. The performance was measured by the AUC values on the test set partition of the patients (n=17).

| **Parameter for predictive model** | **AUC** |
| --- | --- |
| TIS score_*CXCL13*_*CXCL9*_*CD274*_XP cluster 1 score | 0.78 |
| *CXCL9*_*CD8A*_*CD274*_*ERCC2*_XP cluster 1 score | 0.78 |
| *CXCL9*_*CD8A*_*XPC*_*ERCC1*_XP cluster 1 score | 0.78 |
| *CD8A*_*CD274*_*ERCC3*_*ERCC4*_XP cluster 1 score | 0.78 |
| *XPA*_*ERCC2_ERCC1*_XP cluster 1 score_XP cluster 2 score | 0.78 |
| *CXCL9*_*CD8A*_*XPA*_*ERCC2*_*ERCC5* | 0.77 |
| *CXCL9*_*CD274*_*XPA*_*ERCC4*_*ERCC1* | 0.77 |
| *CD8A*_*CD274*_*DDB2*_*ERCC1*_XP cluster 1 score | 0.77 |
| *CD274*_*XPA*_*XPC*_*ERCC4*_*ERCC1* | 0.77 |
| *CD274*_*DDB2*_*ERCC4*_*ERCC1*_XP cluster 1 score | 0.77 |
| TIS score_*CXCL13*_*CD8A*_*ERCC4*_*ERCC1* | 0.75 |
| TIS score_*CD8A*_*CD274*_*ERCC1*_XP cluster 1 score | 0.75 |
| TIS score_*CD8A*_*CD274*_XP cluster 1 score_XP cluster 2 score | 0.75 |
| TIS score_*CD8A*_*ERCC3*_*ERCC4*_XP cluster 1 score | 0.75 |
| TIS score_*CD274*_*ERCC2*_*ERCC4*_XP cluster 1 score | 0.75 |
| *CXCL13*_*CD8A*_*CD274*_*ERCC4*_*ERCC1* | 0.75 |
| *CXCL9*_*CD8A*_*CD274*_*ERCC3*_XP cluster 1 score | 0.75 |
| *CXCL9*_*CD8A*_*XPC*_*ERCC2*_XP cluster 1 score | 0.75 |
| *CXCL9*_*CD274*_*XPA*_*ERCC4*_XP cluster 1 score | 0.75 |
| *CXCL9*_*CD274*_*ERCC3*_*ERCC1*_XP cluster 1 score | 0.75 |
| *CD8A*_*CD274*_*XPC*_*ERCC4*_XP cluster 1 score | 0.75 |
| *CD8A*_*XPA*_*XPC*_*ERCC4*_XP cluster 1 score | 0.75 |
| *CD8A*_*XPC*_*ERCC2*_*ERCC4*_*ERCC1* | 0.75 |
| *CD274*_*XPC*_*ERCC4*_*ERCC1*_XP cluster 2 score | 0.75 |
| *CD8A*_*ERCC2*_*ERCC4*_*ERCC1*_XP cluster 1 score | 0.74 |

## Supplementary Table S11:

Univariate analysis with segregated patients, based on median values of the gene expression or the scores.

|  | beta | HR (95% CI for HR) | wald.test | p.value |
| --- | --- | --- | --- | --- |
| age_situation | -0.55 | 0.58 (0.41-0.8) | 11 | 0.0011 |
| *CD274*_median | 0.52 | 1.7 (1.2-2.3) | 10 | 0.0014 |
| *CD8A*_median | 0.44 | 1.6 (1.1-2.1) | 7.2 | 0.0071 |
| *CXCL9*_median | 0.44 | 1.5 (1.1-2.1) | 7 | 0.008 |
| TIS_score_median | 0.43 | 1.5 (1.1-2.1) | 6.8 | 0.0094 |
| *CXCL13*_median | 0.39 | 1.5 (1.1-2) | 5.8 | 0.016 |
| *ERCC5*_median | 0.29 | 1.3 (0.97-1.8) | 3.1 | 0.078 |
| *ERCC3*_median | 0.25 | 1.3 (0.93-1.8) | 2.3 | 0.13 |
| TMB_median | 0.25 | 1.3 (0.93-1.8) | 2.3 | 0.13 |
| *ERCC2*_median | -0.22 | 0.8 (0.58-1.1) | 1.8 | 0.18 |
| XP cluster 1 score_median | 0.2 | 1.2 (0.88-1.7) | 1.4 | 0.23 |
| *XPA*_median | 0.18 | 1.2 (0.87-1.7) | 1.2 | 0.27 |
| *POLH*_median | -0.18 | 0.84 (0.61-1.2) | 1.2 | 0.27 |
| *XPC*_median | 0.16 | 1.2 (0.86-1.6) | 1 | 0.31 |
| *ERCC4*_median | 0.17 | 1.2 (0.86-1.6) | 1 | 0.31 |
| patient.gender | 0.18 | 1.2 (0.84-1.7) | 1 | 0.31 |
| *ERCC1*_median | 0.11 | 1.1 (0.81-1.5) | 0.46 | 0.5 |
| *DDB2*_median | -0.054 | 0.95 (0.69-1.3) | 0.11 | 0.74 |
| XP cluster 2 score_median | 0.0055 | 1 (0.73-1.4) | 0 | 0.97 |

# Supplementary Figures

# Supplementary Figure S1:

The heatmap of the TCGA cohort’s log_2_ transformed FPKM value of the nine XP genes, partitioned into (A) primary or (B) metastatic samples, (C) young or (D) old, and (E) male or (F) female patients.

**Supplementary Figure S2:**

Correlation of TIS score, the single immune-infiltration genes, the XP genes and the computed cluster scores. The correlation maps illustrated show the TCGA-cohort fractioned by the clinical parameters (A) primary or (B) metastatic samples, (C) younger or (D) older patients, and (E) male or (F) female patients**.**

**Supplementary Figure S3:**

Kaplan-Meier survival curves of patients stratified by the median of (A) age, (B) TIS score, (C) cluster 1 XP score and (D) cluster 2 XP score. The illustrated *p*-values are based on the log-rank test.

**Supplementary Figure S4:**

Results of univariate cox regression analysis of patients of primary samples (n = 95).

**Supplementary Figure S5:**

Results of univariate cox regression analysis of patients of metastatic samples (n = 348).

**Supplementary Figure S6:**

Results of univariate cox regression analysis of male patients (n=275).

**Supplementary Figure S7:**

Results of univariate cox regression analysis of female patients (n=168).

**Supplementary Figure S8:**

Boxplot of ICI response data (n=75), compared with Wilcoxon test based on the expression of (A) XP cluster 2 score, (B) TIS score*,* (C) *CD27* and (D) *PSMB10*.
